# Blood Urea Nitrogen to Serum Albumin Ratio Is Associated with MASH and Significant Fibrosis: Report from a Turkish Biopsy-Proven MASLD Cohort

**DOI:** 10.3390/jcm15155892

**Published:** 2026-07-28

**Authors:** Ali Kirik, Eda Kaya, Caglayan Keklikkiran, Yusuf Yilmaz

**Affiliations:** 1 Department of Internal Medicine, Balikesir University Medical School, Balikesir 10145, Türkiye; alikirik87@hotmail.com; 2 The Global NASH Council (GNC), Washington, DC 20037, USA; eda.kaya@knappschaft-kliniken.de (E.K.); caglayan.keklikkiran@erdogan.edu.tr (C.K.); 3 Department of Medicine, Knappschaft Kliniken University Hospital Bochum, Ruhr-University Bochum, 44892 Bochum, Germany; 4 Department of Gastroenterology, School of Medicine, Recep Tayyip Erdogan University, Rize 53020, Türkiye; 5 Dijitalpark Teknokent, İstanbul 34788, Türkiye

**Keywords:** MASLD, blood urea nitrogen-to-serum albumin ratio, MASH, fibrosis

## Abstract

**Background/Objectives:** The blood urea nitrogen-to-serum albumin ratio (BAR) is a recently established index that has been associated with systemic inflammation. This study aimed to evaluate the association between BAR levels and histopathological findings in patients with biopsy-proven MASLD. **Methods:** A total of 425 biopsy-proven MASLD patients from the Turkish MASLD Biobank were included in the analysis. Histological assessments were performed according to the SAF/FLIP algorithm. The BAR was calculated as indicated. **Results:** The median age was 49 [19–71] and54% of the patients were male (*n* = 229). A total of 204 (48%) and 377 patients (88.7%) had significant fibrosis and metabolic dysfunction-associated steatohepatitis (MASH), respectively. BAR was significantly lower among patients with significant fibrosis [3.11 (0.21–9.41) vs. 3.41 (1.37–11.49), *p* = 0.002]. The same trend was observed among patients with MASH (3.2 [0.2–11.5] vs. 4.6 [1.9–8.7]), *p* < 0.001). Also, there is a decline beginning from F0 to F4 fibrosis (*p* = 0.014). In multivariate analysis, BAR remained an independent predictor after adjustment for confounders (OR 0.813, *p* = 0.020). BAR showed an AUROC of 0.586 (95% CI: 0.532–0.640) for predicting significant fibrosis. **Conclusions:** This is the first study to evaluate BAR levels in patients with biopsy-proven MASLD, demonstrating that BAR levels were inversely associated with the presence of significant fibrosis. These findings show an association between declining BAR levels and impaired liver health. However, the results should be interpreted cautiously owing to their suboptimal statistical performance.

## 1. Introduction

Metabolic dysfunction-associated steatotic liver disease (MASLD), formerly known as nonalcoholic fatty liver disease (NAFLD), is a chronic liver disease directly associated with cardiometabolic risk factors (CMRF) [1]. MASLD is the most common chronic liver disease globally, with a prevalence estimated at 25–30% worldwide [2]. The disease begins with simple steatosis in the early stages and can progress to steatohepatitis and fibrosis over time [3]. The primary causes of this condition are insulin resistance, oxidative stress, and increased systemic inflammation. Indeed, MASLD, a leading cause of liver cirrhosis today, plays a key role in the deterioration of liver health [4].

Blood urea nitrogen (BUN) to serum albumin ratio (BAR) is a novel, defined index that is emphasized to be related to systemic inflammation [5]. BUN is an end product of the protein metabolism pathway and is directly related to kidney and liver function. BUN is synthesized in the liver and excreted through the kidneys; plasma levels of BUN may increase in dehydration, high protein intake, and renal dysfunction, while they may decrease in liver dysfunction. Albumin is the most abundant protein molecule in systemic circulation and plays a key role in the formation of plasma oncotic pressure. Originally synthesized in the liver, albumin is a negative acute phase reactant directly associated with systemic inflammatory diseases. The two components of BAR, BUN and albumin, are directly related to renal dysfunction, systemic inflammation, cardiovascular disease (CVD), malnutrition, impaired liver function, and medical drugs [6,7,8,9]. The classical paradigm suggests an increase in BUN levels and a decrease in albumin levels during systemic inflammation. In light of this data, it is emphasized that BAR, an index that has recently begun to be studied, may be associated with inflammatory processes [10]. Current clinical research highlights that BAR levels may have prognostic value in infectious diseases, CVD, acute pancreatitis, and gastrointestinal bleeding [11,12,13].

Today, the role of many different indices, particularly the neutrophil-to-lymphocyte ratio, in MASLD has been investigated. However, to our knowledge, no study has examined the clinical significance of BAR levels in patients with MASLD. In this study, we aimed to evaluate the relationship between BAR levels and pathological findings in patients with biopsy-proven MASLD.

## 2. Materials and Methods

### 2.1. Study Design and Population

Data for this analysis were obtained from the Turkish MASLD Biobank, which includes demographic, biochemical, and histological information from Turkish patients diagnosed with MASLD who were evaluated at our tertiary care center. Patients with viral hepatitis, drug-induced liver injury, autoimmune hepatitis, metabolic or genetic liver diseases, or significant alcohol consumption (>20 g/day for women and >30 g/day for men) were excluded. The data collection procedures, which were conducted at a tertiary care center using an identical liver biopsy indication strategy, have been described in detail elsewhere [14].

### 2.2. Ethics

This study adheres to the ethical principles for medical research outlined in the Declaration of Helsinki and was approved by the Ethics Committee of the School of Medicine at Recep Tayyip Erdoğan University (date: 4 February 2026; protocol number: 2026/9). Given the retrospective nature of the study, the requirement for informed consent was waived.

### 2.3. Definition of Metabolic Disorders

MASLD was defined as the presence of hepatic steatosis in combination with at least one CMRF in the absence of significant alcohol intake [4]. These risk factors included a body mass index (BMI) ≥ 25 kg/m^2^ or waist circumference (WC) > 94 cm for men and >80 cm for women; fasting plasma glucose (FPG) ≥ 100 mg/dL, a two-hour post-load glucose level ≥ 140 mg/dL, or HbA1c ≥ 5.7%; blood pressure ≥ 130/85 mmHg or use of antihypertensive medication; plasma triglycerides ≥ 150 mg/dL or lipid-lowering therapy; and high-density lipoprotein cholesterol (HDL-C) ≤ 40 mg/dL for men or ≤50 mg/dL for women, or lipid-lowering treatment.

### 2.4. Biochemical Parameters

Laboratory parameters of all patients were evaluated for analysis. Low-density lipoprotein cholesterol (LDL-C) was calculated by the Friedewald formula [LDLC = TC − (HDL-C + TG/5)] [15]. The BAR level was calculated from the ratio of BUN (mg/dL) to albumin (g/dL) [5].

### 2.5. Liver Histology

Liver biopsy was performed in patients with imaging-confirmed hepatic steatosis who had persistently elevated aminotransferase levels for more than six months. In addition, individuals with hepatomegaly or splenomegaly in the presence of normal liver enzyme levels were also considered eligible for biopsy. All histological assessments were conducted by a single experienced pathologist using the Steatosis–Activity–Fibrosis (SAF) scoring system. A diagnosis of metabolic dysfunction-associated steatohepatitis (MASH) was established when a score of at least one was assigned in each of the following domains: steatosis, lobular inflammation, and hepatocellular ballooning; otherwise, MAFL. Fibrosis stage F ≥ 2 was classified as significant fibrosis, while stage F ≥ 3 was considered advanced fibrosis [16].

### 2.6. Statistical Analysis

Categorical variables are presented as frequencies and percentages. Data distribution was evaluated using the Kolmogorov–Smirnov test. Variables with a normal distribution are expressed as mean ± standard deviation, whereas non-normally distributed data are reported as median (range). Comparisons between normally distributed variables were performed using Student’s *t*-test, while non-normally distributed variables were analyzed using the Mann–Whitney U test and the Kruskal–Wallis test, as appropriate. Categorical variables were compared using the chi-square test. Following univariate analysis, binary logistic regression was performed to identify independent predictors of significant fibrosis. Parameters that were statistically significant in the univariate analysis were included in the binary logistic regression model using the ‘Enter’ method. To avoid multicollinearity, clinically overlapping variables, such as blood pressure and a diagnosis of hypertension, were not included simultaneously. Model calibration and goodness-of-fit were assessed using the Hosmer–Lemeshow test. Receiver operating characteristic (ROC) curve analysis was used to evaluate the ability of the BAR to predict the presence of significant fibrosis, and the optimal cut-off value was determined using the Youden index. The performance of BAR was compared with the Fibrosis-4 (FIB-4) index and the AST to platelet ratio (APRI). All analyses were performed using SPSS v.29 for Windows (IBM, Armonk, NY, USA).

## 3. Results

The general characteristics and comparison of demographic and laboratory parameters according to MASH and fibrosis status are depicted in Table 1 and Table 2 [median age: 49 (19–71), 53.9% male (*n* = 229)]. A total of 425 patients with biopsy-proven MASLD [MASH; *n* = 377; median age 50 (19–71)], metabolic dysfunction-associated steatotic liver (MASL) [*n*= 48; median age 46 (23–65)] were enrolled in the analysis. Furthermore, all patients were divided into two groups according to the presence of significant fibrosis, and 204 patients [median age 53 (19–71)] had significant fibrosis. The prevalences of diabetes mellitus (67% vs. 37%) and hypertension (50% vs. 29%) were significantly higher in patients with significant fibrosis compared to those without (*p* < 0.001 for both). In this study cohort, only two patients had a glomerular filtration rate (GFR) <60 mL/min/1.73 m^2^ (54 and 56 mL/min/1.73 m^2^, respectively.

**Figure 1:**
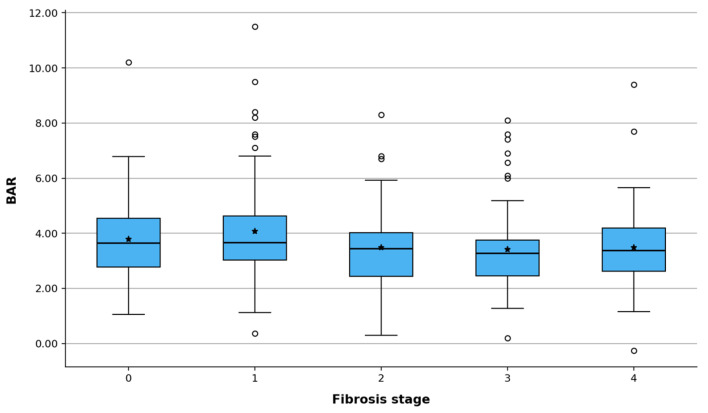
The comparison of the BAR levels according to the fibrosis stage. Circles represent outliers, defined as observations exceeding 1.5 times the interquartile range (IQR) below the first or above the third quartile. Asterisks indicate mean values.

According to the paired comparisons, patients with MASH had significantly lower BAR levels [3.2 (0.2–11.5)] than the MASL group [4.5 (1.9–8.7)] (*p* < 0.001). Additionally, BAR levels were significantly lower among patients with significant fibrosis [3.11 (0.21–9.41) vs. 3.41 (1.37–11.49), *p* = 0.002]. Moreover, BAR showed a significant decline with increasing fibrosis stage (*p* = 0.014) (Figure 1).

**Figure 2:**
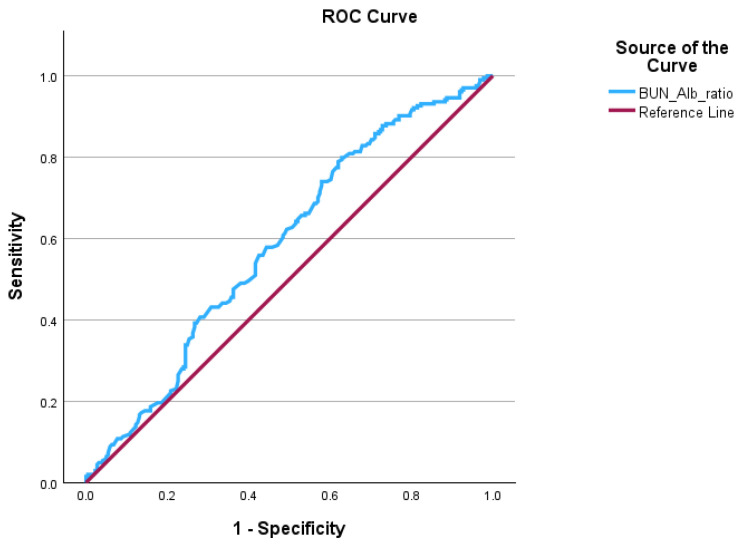
Receiver operating characteristic curves for predicting significant fibrosis. AUROC = 0.586 (95% CI: 0.532–0.640).

In the multivariable binary logistic regression analysis, BAR remained an independent predictor after adjustment for variables that were statistically significant in the univariate analyses [Odds ratio: 0.813, *p* = 0.020] (Table 3). In a separate analysis that included albumin and BUN instead of BAR, BUN remained significantly associated with the outcome (*p* = 0.012), whereas albumin did not. The Hosmer-Lemeshow test showed no evidence of poor model fit (*p* = 0.387). BAR showed an AUROC of 0.586 (95% confidence interval: 0.532–0.640) for predicting significant fibrosis, considering its reverse relationship with significant fibrosis (Figure 2). According to Youden’s index, the best cut-off for identifying significant fibrosis was 4.06. At this threshold, sensitivity was 31%, and specificity was 63%. The positive and negative predictive values were 33% and 60%, respectively. In comparison, FIB-4 (AUROC: 0.658, 95% CI: 0.605–0.712) and APRI (AUROC: 0.732, 95% CI: 0.683–0.780) exhibited superior predictive performance for significant fibrosis.

## 4. Discussion

BAR level is a novel index that has been shown to have prognostic value in many different clinical studies, but its role in the pathogenesis of MASLD is unknown. To our knowledge, the study presented here is the first to demonstrate the role of BAR in patients with MASLD, with BAR levels being higher in participants with MASL compared to the MASH patients. Interestingly, BAR levels show a statistically significant decrease with the increase in fibrosis stage. On the other hand, it should be stated that there was a relatively low difference among the fibrosis stages. Nevertheless, this suggests that a decrease in BAR levels in individuals with MASLD may be associated with liver injury. Conversely, the performance of BAR in predicting significant fibrosis remained suboptimal, limiting its potential clinical utility. Moreover, BAR demonstrated lower diagnostic performance than the established non-invasive fibrosis markers, FIB-4 and APRI.BAR is a novel prognostic indicator that is being investigated in various clinical pathologies, particularly in infectious diseases [17]. In particular, it is thought that increased BAR levels may have prognostic value in inflammatory processes such as gastrointestinal bleeding, cardiovascular diseases, and idiopathic pulmonary fibrosis [13,18,19]. However, the role of BAR is not fully understood in patients with MASLD. Albumin levels, one of the two parameters of this index, are significantly reduced in chronic liver disease [8]. However, the changes in BUN levels in liver pathologies are not fully known. Renal impairment occurring in the course of chronic liver disease causes an increase in BUN levels [20]. On the other hand, conditions such as liver dysfunction, malnutrition, disruption of the urea cycle, and cachexia cause a significant decrease in BUN levels [21,22]. This complicates the role of BUN levels in pathogenesis. Indeed, in the current literature, there are two studies examining the role of BUN in individuals with MASLD, and the clinical results of these studies are inconsistent. A study by Xudong Liu et al. found that BUN levels in individuals with MASLD were statistically significantly higher compared to controls [23]. However, another study by Ercin et al. in patients with biopsy-proven MASLD showed that there was no significant relationship between BUN levels and metabolic, biochemical, and histopathological findings [24].

Although elevated BAR levels are known to be associated with inflammatory diseases, there are insufficient studies investigating the role of BAR in chronic liver diseases. A clinical study by Shen et al. found that elevated BAR was associated with poor outcomes in patients with hepatitis B virus-related decompensated cirrhosis [25]. However, there are no clinical studies in the current literature investigating the role of BAR in patients with MASLD. In this study, we found statistically significantly lower BAR levels in patients with the MASH group compared to those with the simple steatosis group. Furthermore, lower BAR levels were observed in the group with significant fibrosis compared to the group without significant fibrosis. Furthermore, the significant decrease in BAR levels in the fibrosis subgroup analysis is another noteworthy point. This suggests that a decrease in BAR levels may be an early warning factor for liver health impairment in individuals with MASLD.

BAR level is a laboratory indicator derived from two paradigms of biological and clinical significance [5]. In clinical use, it is frequently associated with inflammatory diseases, and its role in liver diseases is unclear [13,18,19]. In patients with MASLD, plasma albumin levels tend to decrease as fibrosis and cirrhosis progress. On the other hand, BUN levels are variable during the development of chronic liver disease. While malnutrition and urea cycle disorders cause a decrease in BUN levels, conditions such as hepatorenal syndrome cause an increase in plasma BUN levels. Indeed, this variability in BUN levels makes it difficult to understand the role of BAR levels in chronic liver disease. In light of this data, in this study we observed that low BAR levels in patients with MASLD may be associated with fibrosis stages. However, our findings have a weak effect size compared to traditional fibrosis scoring systems and need to be supported by further studies. Furthermore, BAR levels are based on a mathematical measurement, and further studies are needed to elucidate their direct relationship with pathophysiological mechanisms.

In our study, the optimal cut-off yielded a moderate performance with a higher specificity. In conjunction with the low AUROC, these findings indicate that the BAR has poor discriminative performance for identifying significant fibrosis. Although several noninvasive tests are primarily valued for their ability to exclude advanced fibrosis and are therefore widely used as first-line triage tools in MASLD [26,27,28], our results suggest that BAR is not suitable for this purpose. Instead, BAR may be more appropriately interpreted as an indirect marker of disease severity, showing an inverse relationship with fibrosis severity.

The major strength of our study is that it was designed using clinical data from a large cohort of patients with biopsy-proven MASLD. However, our study has several limitations. Firstly, the cross-sectional design of the study cannot fully establish the causality between BAR and the pathological process. Therefore, statistically significant associations may not necessarily indicate causality. Moreover, since the data were derived from a tertiary care center, selection bias cannot be excluded, which limits the generalizability of these results to the broader population. Secondly, the history of medication and nutritional supplement use that could potentially affect BAR levels was not evaluated due to a lack of available data. Thirdly, the patients’ past BAR levels and changes over time could not be analyzed. Furthermore, there were only two patients with slightly lower glomerular filtration rates in our study, indicating that the cohort generally consisted of individuals with normal kidney function. Consequently, the real-world applicability of these findings should be considered carefully. Since impaired kidney function would further affect the BAR score, its clinical utility must be evaluated with caution in patients with renal impairment. Lastly, the incremental diagnostic value of BAR appears to be limited, as established non-invasive fibrosis markers, including FIB-4 and APRI, demonstrated superior diagnostic performance.

## 5. Conclusions

In conclusion, BAR is a novel marker that evaluates plasma albumin and BUN levels together, and its role in patients with MASLD is unknown. This is the first study to examine BAR levels in patients with biopsy-proven MASLD, and BAR levels showed a decrease with increasing fibrosis stage. It is suggested that decreased BAR levels during clinical follow-up may be an early indicator of impaired liver health. However, its clinical implementation remains limited due to its low performance, and it must be interpreted cautiously. Further clinical research is crucial to understand the role of BAR in the pathogenesis of MASLD.

## Figures and Tables

**Table 1:** Comparison of clinical and biochemical parameters of patients with MASH and MASL groups.

Parameter	Total Group (*n* = 425)	MASH Group (*n* = 377)	MASL Group (*n* = 48)	*p* Value
Age, years	49 [19–71]	50 [19–71]	46 [23–65]	**0.016**
Sex, male	229 (53.9%)	196 (52.0%)	33 (68.8%)	**0.028**
BMI, kg/m2	32.0 [23.1–56.0]	32.2 [23.1–56.0]	30.5 [23.3–43.1]	**0.008**
SBP, mmHg	128 [100–190]	128 [100–190]	120 [100–152]	**<0.001**
DBP, mmHg	82 [51–120]	83 [51–120]	80 [60–100]	0.115
WC, cm	105 [70–147]	106 [70–141]	102 [83–147]	**0.007**
HC, cm	109 [63–155]	109 [63–155]	107 [89–142]	0.124
AST, U/L	40 [11–168]	41 [11–168]	33.5 [12–73]	**<0.001**
ALT, U/L	64 [12–252]	66 [12–252]	48.5 [12–179]	**0.001**
ALP, U/L	82 [11–226]	82 [34–190]	79.5 [11–226]	0.863
GGT, U/L	48 [8–251]	49 [8–251]	37 [12–182]	**0.010**
LDH, U/L	205 [15–449]	206 [15–449]	201 [135–449]	0.182
Total bilirubin, mg/dL	0.7 [0.05–3.4]	0.7 [0.1–3.4]	0.7 [0.2–2.4]	0.941
Albumin, g/dL	4.6 [3.4–5.9]	4.6 [3.4–5.9]	4.7 [3.8–5.1]	0.153
Total cholesterol, mg/dL	201 [74–419]	201 [74–417]	195 [128–419]	0.242
Triglycerides, mg/dL	160 [31–1107]	160 [31–1107]	152 [61–453]	0.973
HDL-C, mg/dL	44 [5–84]	45 [19–84]	41 [5–77]	0.178
LDL-C, mg/dL	127 [15–400]	127 [15–400]	126 [73–218]	0.904
FPG, mg/dL	103 [66–342]	104 [66–342]	98 [70–261]	**0.010**
HbA1c, %	5.9 [4–11.1]	6 [4–11.1]	5.6 [4.8–11–1]	**0.005**
TSH	1.6 [0.02–131]	1.6 [0.02–131]	1.6 [0.3–9.37]	0.960
Plt	235 [77–543]	233 [77–543]	244 [90–475]	0.107
Uric acid	6.1 [0.90–11]	6.1 [0.9–11]	6.5 [2.1–8.3]	0.578
BUN, mg/dL	15 [0.9–54]	14 [0.9–54]	21.5 [9–40]	**<0.001**
Creatinine, mg/dL	0.8 [0.34–1.3]	0.8 [0.3–1.3]	0.9 [0.5–1.2]	**<0.001**
Glomerular filtration rate, mL/min/1.73 m2	104 [54–149]	106 [54–149]	100 [72–121]	**0.021**
BAR	3.3 [0.21–11.49]	3.2 [0.2–11.5]	4.5 [1.9–8.7]	**<0.001**

Abbreviations: BMI, body mass index; SBP, systolic blood pressure; DBP, diastolic blood pressure, WC, waist circumference; HC, hip circumference; AST, aspartate aminotransferase; ALT, alanine aminotransferase; ALP, alkaline phosphatase; GGT, gamma-glutamyl-transferase; LDH, lactate dehydrogenase; HDL-C, high-density-lipoprotein cholesterol; LDL-C, low-density-lipoprotein cholesterol; FPG, fasting plasma glucose; HbA1c, glycated haemoglobin; TSH, thyroid-stimulating hormone; Plt, platelet; BUN, blood urea nitrogen; BAR, blood urea nitrogen to serum albumin ratio. The statistically significant *p*-values are marked in bold.

**Table 2:** Comparison of clinical and biochemical parameters of patients with and without significant fibrosis.

Parameter	Total Group (*n* = 425)	with ≥F2 Fibrosis (*n* = 204)	without ≥F2 Fibrosis (*n* = 221)	*p* Value
Age, years	49 [19–71]	53 [19–71]	43 [22–65]	**<0.001**
Sex, male	229 (53.9%)	94 (46.1%)	135 (61.1%)	**0.002**
BMI, kg/m2	32.0 [23.1–56.0]	32.47 [23.26–55.96]	31.53 [23.12–49.12]	**0.023**
SBP, mmHg	128 [100–190]	130 [100–190]	125 [100–180]	**<0.001**
DBP, mmHg	82 [51–120]	83 [59–115]	80 [51–120]	0.154
WC, cm	105 [70–147]	108 [70–141]	104 [79–147]	**0.002**
HC, cm	109 [63–155]	110 [63–155]	108 [89–143]	0.264
AST, U/L	40 [11–168]	43.9 [11–162]	36 [12–168]	**<0.001**
ALT, U/L	64 [12–252]	66 [13–252]	63 [12–245]	0.384
ALP, U/L	82 [11–226]	86 [34–190]	78 [11–226]	**0.032**
GGT, U/L	48 [8–251]	55.35 [13–251]	42 [8–228]	**<0.001**
LDH, U/L	205 [15–449]	208 [15–449]	202 [19–449]	0.100
Total bilirubin, mg/dL	0.7 [0.05–3.4]	0.70 [0.05–3.40]	0.64 [0.12–2.80]	0.087
Albumin, g/dL	4.6 [3.4–5.9]	4.5 [3.4–5.9]	4.6 [3.7–5.58]	**0.008**
Total cholesterol, mg/dL	201 [74–419]	198 [74–360]	205 [87–419]	0.190
Triglycerides, mg/dL	160 [31–1107]	170 [31–584]	150 [44–1107]	0.122
HDL-C, mg/dL	44 [5–84]	44.9 [19–84]	44 [5.56–82]	0.865
LDL-C, mg/dL	127 [15–400]	122 [15–400]	131 [40–400]	**0.020**
Glucose, mg/dL	103 [66–342]	113 [70–342]	99 [66–270]	**<0.001**
HbA1c, %	5.9 [4–11.1]	6.2 [4.1–11.1]	5.7 [4.0–11.1]	**<0.001**
TSH	1.6 [0.02–131]	1.64 [0.02–131]	1.538 [0.22–22.08]	0.338
Plt	235 [77–543]	224 [77–410]	244 [89–543]	**<0.001**
Uric acid	6.1 [0.90–11]	6.05 [0.90–11.00]	6.2 [2.10–9.80]	0.592
BUN, mg/dL	15 [0.9–54]	14 [0.91–39]	15 [7–54]	**<0.001**
Creatinine, mg/dL	0.8 [0.34–1.3]	0.760 [0.34–1.30]	0.835 [0.48–1.29]	**<0.001**
Glomerular filtration rate, mL/min/1.73 m2	104 [54–149]	105 [57–143]	104 [54–149]	0.947
BAR	3.3 [0.21–11.49]	3.11 [0.21–9.41]	3.41 [1.37–11.49]	**0.002**

Abbreviations: BMI, body mass index; SBP, systolic blood pressure; DBP, diastolic blood pressure, WC, waist circumference; HC, hip circumference; AST, aspartate aminotransferase; ALT, alanine aminotransferase; ALP, alkaline phosphatase; GGT, gamma-glutamyl-transferase; LDH, lactate dehydrogenase; HDL-C, high-density-lipoprotein cholesterol; LDL-C, low-density-lipoprotein cholesterol; FPG, fasting plasma glucose; HbA1c, glycated haemoglobin; TSH, thyroid-stimulating hormone; Plt, platelet; BUN, blood urea nitrogen; BAR, blood urea nitrogen to serum albumin ratio. The statistically significant *p*-values are marked in bold.

**Table 3:** Binary logistic regression analysis for significant fibrosis.

Variables in the Equation								95% Confidence Interval	
		B	S.E.	Wald	df	Sig.	Exp(B)	Lower	Upper
**Step 1 ^a^**	**Age**	0.057	0.014	17.053	1	**<0.001**	1.058	1.031	1.088
	**BMI**	0.044	0.026	2.930	1	0.087	1.045	0.994	1.100
	**AST**	0.012	0.005	5.658	1	**0.017**	1.012	0.986	1.023
	**ALP**	−0.005	0.005	1.069	1	0.301	0.995	0.986	1.005
	**GGT**	0.010	0.003	8.730	1	**0.003**	1.010	1.003	1.017
	**LDL-C**	−0.005	0.003	3.255	1	0.071	0.995	0.990	1.000
	**Creatinine**	−1.924	0.937	4.214	1	**0.040**	0.146	0.023	0.917
	**Plt**	−0.007	0.002	12.676	1	**<0.001**	0.993	0.989	0.997
	**Gender**	−0.222	0.319	0.484	1	0.486	1.248	0.668	2.331
	**DM**	0.515	0.260	3.928	1	**0.047**	1.673	1.006	2.784
	**HT**	−0.178	0.267	0.445	1	0.505	0.837	0.496	1.413
	**BAR**	−0.208	0.089	5.416	1	**0.020**	0.813	0.682	0.968
	**Constant**	−0.646	1.521	0.181	1	0.671	0.524		

^a^. Variable(s) entered on step 1: Age, BMI, AST, ALP, GGT, LDL-C, Creatinine, Plt, Gender, DM, HT, BAR. Abbreviations: BMI, body mass index; AST, aspartate aminotransferase; ALP, alkaline phosphatase; GGT, gamma-glutamyl-transferase; LDL-C, low-density-lipoprotein cholesterol; Plt, platelet; DM, diabetes mellitus; HT, hypertension; BAR, blood urea nitrogen to serum albumin ratio. The statistically significant *p*-values are marked in bold.

## Data Availability

The datasets used and analyzed during the current study are available from the corresponding author upon reasonable request.
